# A Complicated Peptic Ulcer With Bleeding, Gastric Outlet Obstruction, and Choledochoduodenal Fistula

**DOI:** 10.7759/cureus.11189

**Published:** 2020-10-26

**Authors:** Tek N Yadav, Kunal Bikram Deo, Sujan Gautam, Laligen Awale, Narendra Pandit

**Affiliations:** 1 Surgery, B.P. Koirala Institute of Health Sciences, Dharan, NPL; 2 Surgery/Surgical Gastroenterology, B.P. Koirala Institute of Health Sciences, Dharan, NPL

**Keywords:** complicated peptic ulcer disease, bleeding, gastric outlet obstruction, choledochoduodenal fistula, surgery

## Abstract

Although peptic ulcer disease (PUD) is a common entity, the rate of its complication has decreased with the advent of proton pump inhibitors. We present a case of complicated PUD in a 49-year-old male patient having a rare combination of bleeding, gastric outlet obstruction, and a large choledochoduodenal fistula (CDF) who presented with shock. After resuscitation and investigations, ligation of bleeder via duodenotomy, Roux-en-Y choledochojejunostomy, and gastrojejunostomy was done for ulcer bleeding, CDF, and pyloric stenosis respectively. The patient improved after surgery. As with other emergency surgery, minimizing morbidity and mortality remains the principle of management. The best treatment in this situation irrespective of hemodynamic stability is surgery, which is a one-time and best treatment for bleeding, obstruction, and CDF.

## Introduction

Peptic ulcer disease (PUD) is a common entity with the potential to cause complications such as bleeding (19 to 57 cases per 100,000 individuals), perforation (four to 14 cases per 100,000 individuals), obstruction, and rarely bilio-enteric fistula [1]. However, with the advent of proton pump inhibitors, the rate of complications has decreased [2]. Moreover, the presence of a combination of multiple complications of peptic ulcer in the index case is very rare to observe in the modern era and is often challenging to manage. We present a case of complicated PUD with a rare combination of bleeding, gastric outlet obstruction, and choledochoduodenal fistula (CDF).

## Case presentation

**Figure 1:**
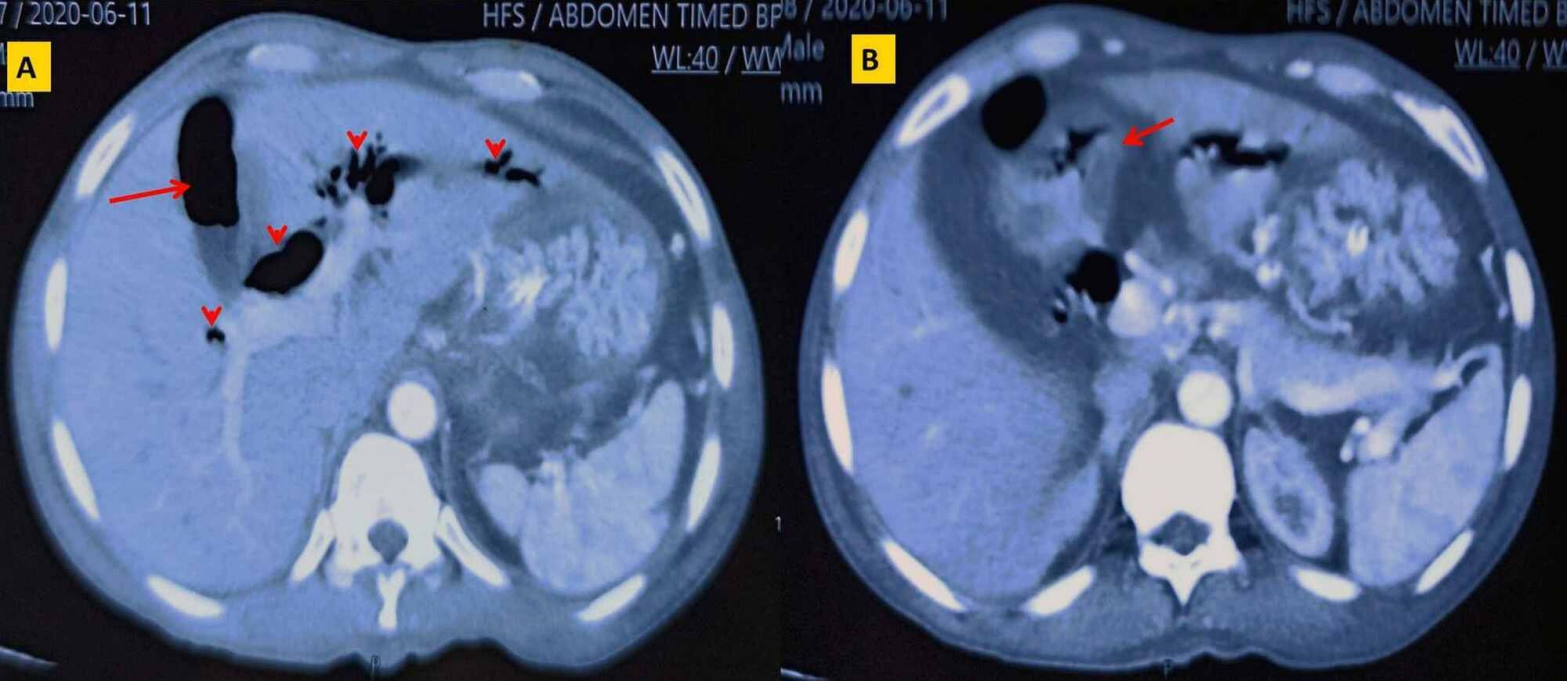
Contrast enhanced computed tomogram (CECT) of the abdomen CECT of the abdomen showing A: extensive pneumobilia seen in the gall bladder *(red arrow)* and both the intrahepatic and hilar bile ducts *(red arrow heads)*. B: thickened duodenal wall in the first part *(red arrow).*

A 49-year-old male, a smoker with no known co-morbidities, presented to the emergency department with a history of melena for seven days. It was associated with episodes of syncopal attacks and generalized weakness. On further inquiry, he had a history of belching and dyspeptic symptoms for the last six years. The patient also gave a history of vomiting (on/off) for the past four months which was projectile, voluminous, and non-bilious. He denied any history of fever, jaundice, abdominal pain, anorexia, and significant weight loss. On general physical examination, the patient was pale, tachycardic (pulse rate = 126 bpm), and hypotensive (blood pressure = 80/60 mm of Hg). The abdominal examination was unremarkable apart from melena on digital rectal examination. His hemoglobin was 6.8 gm/dl, while the remaining blood investigations were essentially normal. His vitals parameter normalized after resuscitation with intravenous fluids and three units of packed cell blood transfusion. Upper gastrointestinal (GI) endoscopy revealed multiple ulcers in the antrum with deformed pylorus and the scope was not negotiable into the duodenum. However, there was no active bleeding from the stomach. The contrast-enhanced computed tomogram (CECT) of the abdomen and pelvis revealed a hugely distended stomach, distorted pylorus (without mass lesion), slightly symmetrical, thickened first part of the duodenum (11 mm) with extensive pneumobilia involving the gallbladder and both the lobes of the liver (Figure 1). Moreover, the abdominal ultrasonogram excluded the stone disease of the gallbladder or the bile duct.

The patient received the diagnosis of complicated PUD in the form of bleeding, gastric outlet obstruction, and the CDF. Emergency surgery was done, which revealed a distended stomach with a deformed and thickened first part of the duodenum (1 cm). There were no features suggestive of gastric malignancy. The longitudinal duodenotomy (2 cm) in the first part revealed a posterior ulcer (2 cm size) with a bleeder and a 1x1 cm CDF on its superior aspect. Four-quadrant suture ligation of the ulcer bleed was done. The duodenotomy was closed transversely with interrupted 4-0 polydioxanone suture (PDS). An additional cholecystectomy and Roux-en-Y choledochojejunostomy (side-to-side), interrupted 4-0 PDS was performed for biliary drainage. The gastric outlet obstruction was managed with posterior, dependent retro-colic gastrojejunostomy. Furthermore, truncal vagotomy and feeding jejunostomy was done. The patient was managed in an intensive care unit (ICU) where inotropic support was gradually tapered off over the next three days. He also developed hospital-acquired pneumonia in the immediate postoperative period and recovered well. At the three-month follow-up, the patient is doing well.

## Discussion

This is an interesting case of complicated PUD with a rare combination of three complications, namely bleeding, gastric outlet obstruction, and CDF. The novelty in this report is encountering these three complications in a single patient with PUD, which is rare nowadays. It is further noteworthy, as it poses a diagnostic as well as a therapeutic challenge, more so in a hemodynamically unstable condition because of the rarity and lack of large studies on its management in such a scenario. This benign disease can very well mimic malignancy as well. Management of such patients requires tackling all three complications in the same setting. To the best of our knowledge, there are only a few reports of PUD with a combination of all three complications in literature. In the current era of effective acid-reducing pharmacotherapies, the rate of complications has decreased but the mortality has remained the same. The highest mortality is due to perforation (20%) followed by bleeding (5-10%) [2].

Bleeding is the most common (87%) presentation of complicated PUD which results due to erosion of the underlying gastroduodenal artery and gastric outlet obstruction which accounts for 2.4% of complicated PUD is due to the inflammatory process, pyloric spasm, local edema, scarring, and fibrosis of the involved tissue [2-4]. Emergency endoscopy is the first-line management for both assessment and therapeutic management of bleeding in PUD [5]. However, due to the presence of pyloric stenosis, bleeding duodenal ulcer could not be visualized in our case. In such condition CECT abdomen preferably with angiography is the first-line investigation of choice [5]. Angiography helps in the confirmation as well as localization of the point of hemorrhage and allows treatment by embolization. In addition to diagnosis, this would also help us to plan a trans-catheter arterial embolization (TAE) which has a technical success rate of 92% and a clinical success rate of 72% [5,6]. This may help to avoid morbidity and mortality associated with emergency surgery in an unstable patient and allows sufficient time for resuscitation and further surgical planning. However, the facility of TAE is not commonly available, especially in resource constraint hospitals like ours. Endoscopic balloon dilatation of pyloric stenosis can be tried for gastric outlet obstruction with a long term success rate of 70% - 80% [7,8]. But this requires multiple sessions of dilatation to maintain long term efficacy. Moreover, the presence of an active bleeder in the index case does not allow enough time for dilatation and endoscopic management of the bleeder.

The additional finding of CDF in a patient with peptic ulcer bleeding with pyloric stenosis creates a major dilemma for surgical decision. CDF in PUD is a rare complication in the modern era, occurring due to duodenal ulcer penetrating the common bile duct [9]. While gall stones account for the majority of CDF (73.76%), PUD remains etiology in only 3.16% of all CDF [9]. The reported incidence of CDF by Jaballah et al. [10] was two out of 300 surgeries for duodenal ulcers. CDF in ulcer disease may remain asymptomatic or masked by symptoms of ulcer disease [10]. Presence of pneumobilia, oral contrast in the biliary system indicates the presence of CDF in cross-sectional imaging like CECT abdomen [9]. Cross-sectional imaging also helps to evaluate the possibility of malignancy as well [9]. The surgeon may encounter unexpected findings of CDF during surgery as many times cross-sectional imaging may not be done in an emergency setting. The major concerns of CDF are ascending biliary infections and possibly future biliary stricture [9]. Although small CDF can be managed by medical management and endoscopic management [11], surgical management of larger CDF is preferred [9,12]. Surgical management can be tailored based upon size where CDF less than 0.5 cm can be managed non-surgically, CDF of 0.5 cm to 1 cm requires biliary drainage and >1 cm requires transection of CBD in addition to biliary drainage to prevent reflux of duodenal juice [9,12]. We opted for biliary drainage as the size of CDF was 1 cm and underrunning of bleeding ulcer bed suture might have incorporated the distal bile duct with consequent narrowing. We also did truncal vagotomy for acid reduction. Gatrojejunostomy was done to relieve obstruction and for diversion of gastric content away from the fistula. In recent times of advanced therapeutic endoscopy, endoscopic evaluation and management of CDF remain the preferred approach as per the availability [13]. But in the index patient, where bleeding required urgent surgical management, pyloric stenosis and CDF can undergo single-stage management in the same setting. We didn’t consider resectional surgery like pancreaticoduodenectomy as described in previous report [14] or gastrectomy as the risk of morbidity and mortality associated with it in an unstable patient can be unacceptably high. Another less morbid option could be T tube drainage of CBD. However, the possible risk of biliary stricture and the need for second surgery would always remain. 

## Conclusions

Complicated PUD with a combination of ulcer bleeding, outlet obstruction, and choledochoduodenal fistula is a rare presentation. As with other emergency surgery, minimizing morbidity and mortality remains the principle of management. The best treatment in this situation, irrespective of the hemodynamic stability, is surgery, which is a one time and best treatment for bleeding, obstruction, and bilio-enteric fistula.
